# Zuo Jin Wan, a Traditional Chinese Herbal Formula, Reverses P-gp-Mediated MDR *In Vitro* and *In Vivo*

**DOI:** 10.1155/2013/957078

**Published:** 2013-03-04

**Authors:** Hua Sui, Xuan Liu, Bao-Hui Jin, Shu-Fang Pan, Li-Hong Zhou, Nikitin Alexander Yu, Jie Wu, Jian-Feng Cai, Zhong-Ze Fan, Hui-Rong Zhu, Qi Li

**Affiliations:** ^1^Department of Medical Oncology, Shuguang Hospital, Shanghai University of Traditional Chinese Medicine, Shanghai 201203, China; ^2^Interventional Cancer Institute of Integrative Medicine & Putuo Hospital, Shanghai University of Traditional Chinese Medicine, Shanghai 200062, China; ^3^Department of Biomedical Sciences, Cornell University, Ithaca, NY 14850, USA; ^4^Department of Molecular Oncology, H. Lee Moffitt Cancer Center and Research Institute, Tampa, FL 33612, USA; ^5^Department of Chemistry, University of South Florida, Tampa, FL 33620, USA

## Abstract

Zuo Jin Wan (ZJW), a typical traditional Chinese medicine (TCM) formula, has been identified to have anticancer activity in recent studies. In this study, we determined the underlying mechanism of ZJW in the reversal effect of multidrug resistance on colorectal cancer *in vitro* and *in vivo*. Our results showed that ZJW significantly enhanced the sensitivity of chemotherapeutic drugs in HCT116/L-OHP, SGC7901/DDP, and Bel/Fu MDR cells. Moreover, combination of chemotherapy with ZJW could reverse the drug resistance of HCT116/L-OHP cells, increase the sensitivity of HCT116/L-OHP cells to L-OHP, DDP, 5-Fu, and MMC *in vitro*, and inhibit the tumor growth in the colorectal MDR cancer xenograft model. ICP-MS results showed that ZJW could increase the concentration of chemotherapeutic drugs in HCT116/L-OHP cells in a dose-dependent manner. Furthermore, we showed that ZJW could reverse drug resistance of colorectal cancer cells by decreasing P-gp level *in vitro* and *in vivo*, which has been represented as one of the major mechanisms that contribute to the MDR phenotype. Our study has provided the first direct evidence that ZJW plays an important role in reversing multidrug resistance of human colorectal cancer and may be considered as a useful target for cancer therapy.

## 1. Introduction

Multidrug resistance (MDR), a phenomenon that has been recognized for several decades, remains one of the primary obstacles to successful cancer therapy [1]. It is defined as resistance to one drug accompanied by resistance to other drugs that may have different structures and mechanisms of action. The overexpression of ATP-binding cassette (ABC) transporters on the membrane of cancer cells, which generate energy from ATP hydrolysis to actively extrude a variety of compounds across the membrane, is one of the best characterized mechanisms for this phenomenon [2]. As far as we know, the ABC transporter-subfamily B member 1 (ABCB1/MDR1/P-glycoprotein, P-gp), subfamily C member-1/2 (ABCC-1/2, MRP-1/2), subfamily G member 2 (ABCG2, BCRP), and lung resistance-related protein (LRP) play a major role in producing MDR in tumor cells.

P-glycoprotein (P-gp), a membrane-associated protein typically found on the plasma membrane, has been studied for comparatively long time (35 years) and many facts are gathered in this field. It has been unequivocally proven that the lower intracellular drug accumulation and MDR phenotype are associated with the elevated expression of P-gp, which is a large transmembrane glycoprotein with molecular weight approximately 170 kD [3]. Functioning as a “barrier,” P-gp transport acts are by reduction in intracellular chemical concentration via active efflux against concentration gradients at the plasma membrane.

BCRP is known to be located in the canalicular membrane of hepatocytes and the apical membrane of kidney proximal tubule cells and has the potential to increase biliary and urinary elimination of xenobiotics that are BCRP substrates [4].

The MRP family is now composed of 9 related ABC transporters that are able to transport structurally diverse lipophilic anions and function as drug efflux pumps [5]. MRP1 is a major active transporter of glutathione, glucuronate, and sulfate conjugates and a resistance factor for anthracyclines, epipodophyllotoxins, vinca alkaloids, and camptothecins [6]. MRP-2 (ABCC2), a ubiquitously expressed ATP-binding cassette transporter, has previously been implicated in cellular efflux of cisplatin [7]. Interestingly, clinical evidence exists indicating that common ABCC2 polymorphisms may affect disposition or efficacy of drugs which are known ABCC2 substrates [8].

Lung resistance protein (LRP) is a member of the vault proteins involved in MDR. LRP has been shown to shuttle anthracyclines out of the nucleus [9]. Some conclusive data showed that the expressions of LRP in patients with gastric cancer without prior chemotherapy are high, indicating that innate drug resistance may exist in gastric cancer [10].

Traditional Chinese medicines (TCMs) have been used as medicines or health supplements in China over thousands of years. Traditional Chinese prescriptions and formulae, which are based on TCM principles, have been also identified to be an effective anti-cancer drugs in cancer patients, such as breast carcinoma [11], gastric cancer [12], and colorectal cancer [13]. Compared with chemotherapeutic drugs, which were defined as the major drug for a long time, traditional Chinese prescriptions and formulae are a therapy to effectively control cancer progression, improve quality of life, and prolong survival times.

Zuo Jin Wan (ZJW), a typical TCM formula, consists of the Rhizoma Coptidis and Fructus Evodiae in the ratio of 6 : 1 (w/w). *Coptis chinensis* Franch, the main component of Rhizoma Coptidis, has recently been reported to have the effect of reversing MDR [14, 15]. Fructus Evodiae has been demonstrated to induce some cancer cell apoptoses, such as human melanoma A375-S2 cells [16], human hepatocellular carcinoma SMMC-7721 cells [17], and Human colorectal carcinoma COLO-205 cells [18]. In addition, previous studies have shown that ZJW can lessen the degree of treating abdominal pain, acid regurgitation, nausea, and so on and enhance the immune function of patients in the gastric ulcer therapy [19].

Although ZJW herbal formula had been found to have an anti-cancer effect, the underlying mechanisms remain unknown. In this study, our objective was to elucidate the effect and the molecular mechanism of Chinese herbs formula ZJW herbal formula in human cancer cells both* in vitro* and *in vivo.*

## 2. Materials and Methods 

### 2.1. Preparation of the Extracts for ZJW

ZJW was formulated by Rhizoma Coptidis and Evodia (in a ratio of 6 : 1). All the herbs were purchased from Putuo Hospital herb store. The mixture (70 g) was extracted twice for 1 h each time by refluxing in ethanol (1 : 8, v/v). The filtrates were concentrated and dried in vacuum at 60°C. The concentrated extract was then dried by lyophilization to obtain the ZJW extract at a yield of dried powder of 24.4%. The extract was stored at 4°C, and its preparations were standardized, regulated, and quality controlled according to the guidelines defined by Chinese State Food and Drug Administration (SFDA).

### 2.2. Cell Culture and Reagents

The human colorectal cancer HCT116 parental cell, gastric cancer SGC7901 cell, and hepatic carcinoma Bel7402 parental cell were purchased from the Shanghai Cell Collection (Shanghai, China). HCT116/L-OHP MDR cell lines were established and maintained in our laboratory; SGC7901/DDP MDR cell line and Bel/Fu MDR cell lines were obtained from Keygen Biotech Co., Ltd. (Nanjing, China). The cells were grown in RPMI 1640 medium supplemented with 10% (v/v) heat-inactivated fetal calf serum, 2 mM glutamine, 100 units/mL penicillin, and 100 *μ*g/mL streptomycin (Invitrogen, Carlsbad, CA, USA) at 37°C in a 5% CO_2_ humidified atmosphere. HCT116/L-OHP cells were routinely maintained in a medium containing 5,000 ng/mL Oxaliplatin (L-OHP), SGC7901/DDP cells were routinely maintained in a medium containing 1000 ng/mL Diamminedichloroplatinum (DDP), and Bel/Fu cells were routinely maintained in a medium containing 2000 ng/L. Mitomycin (MMC) and L-OHP were purchased from Shenzhen Main Luck Pharmaceuticals Co., Ltd. (Shenzhen, China), DDP was purchased from Qilu Pharmaceutical Co., Ltd. (Shandong, China), and 5-Fu was purchased from Shanghai Xudong Haipu Pharmaceutical Co., Ltd. (Shanghai, China). Monoclonal antibodies against ABCB1, ABCC-1/2, BCRP, and LRP and dehydrogenase (GAPDH) antibodies were the products of Cell Signaling Technology (Beverly, MA, USA).

### 2.3. Animals and Xenograft Model

Male athymic nude mice (NCr-nu) were purchased from Sino-British SIPPR/BK lab Animal Co., Ltd (Shanghai, China, license no. SCXK 2003-0002), and maintained under specific pathogen-free conditions for the studies. All animal protocols were approved by the Institutional Animal Use and Care Committee. All the experiments and animal care were approved by Shanghai Medical Experimental Animal Care Commission and in accordance with the Provision and General Recommendation of Chinese Experimental Animals Administration Legislation. The mice used in these experiments were 8- to 12-week old.

HCT116/L-OHP cells were grown in culture and then detached by trypsinization, washed, and resuspended in Hanks (HBSS). 0.2 mL HBSS with cells (1.0 × 10^6^) were subcutaneously injected into the athymic nude mice to initiate tumor growth. When the tumors were allowed to reach an average size of 100 mm^3^, the mice were randomized into 6 groups (*n* = 8 per group). Mice in group 1 were administered with distilled water daily that served as vehicle control. Mice in groups 2–5 were given oxaliplatin as an intraperitoneal injection every two days and the injection dosage (5 mg/kg) was according to half of the maximum tolerated dose (MTD) of oxaliplatin as previously described [20]. Mice in groups 3, 4, and 5 received intragastric administration of ZJW at the doses of 1027.5 mg/kg, 2055 mg/kg, and 4110 mg/kg. In the clinical practice of the Chinese herbal medicine, ZJW is usually prescribed at a daily dose of 10000 mg of herbal materials. When this human dose was converted into an animal dose (a person of 60 kg, and a conversion factor of 12.33 between human and mouse), it was equivalent to the middle dose (2055 mg/kg) used in this study. Mice in group 6 only received intragastric administration of ZJW at the doses of 1027.5 mg/kg, which used as excluding evodiamine toxicity group.

The body weight of the animals and the two perpendicular diameters (*A* and *B*) were recorded every 3 days and the tumor volume (*V*) was estimated according to the following formula [21]: *V* = *π*/6 × [(*A*+*B*)/2]^3^. The curve of tumor growth was drawn according to tumor volume and time of implantation. 6 mice were sacrificed in each group on 28th day after treatment; the other 6 mice in the same group were observed for the survival time. Tumor tissues were excised from the mice and their weight was measured. The time of survival for each group and overall significance were plotted on a Kaplan-Meier survival curve also using GraphPad Prism.

### 2.4. Immunohistology Analysis Was Carried Out Using Paraffin Section

Paraffin section was incubated in a blocking solution (10% donkey serum +5% nonfat dry milk +4% BSA +0.1% Triton X-100) for 10 min and then hydrated sections were incubated at 4°C overnight with anti-P-gp antibody. After washing with phosphate-buffered solution (PBS), the sections were incubated with diluted (1 : 200) biotinylated secondary antibody for 30 min. Subsequently, the slides were washed again in PBS and incubated for 30 min with the preformed avidin-horseradish peroxidase macromolecular complex. Development of peroxidase reaction was achieved by incubation in 0.01% 3,3-diaminobenzidine tetrahydrochloride (DAB) in PBS containing 0.01% hydrogen peroxide for approximately 5 min at room temperature. Sections were then washed thoroughly in tap water, counterstained in haematoxylin, dehydrated in absolute alcohol, cleared in xylene, and mounted in synthetic resin for microscopic examination.

### 2.5. Cell Viability Assays

Cell proliferation was determined using the cell counting kit-8 (CCK-8). Briefly, the cells were seeded in 96-well plates at 1 × 10^4^ cells/well. When the cells reached 60% confluence, the medium was removed and replaced with fresh medium containing varying concentrations of ZJW or its admixture with antitumor drug (L-OHP, DDP, 5-FU and MMC) and incubated for 48 hours. The CCK-8 assay was performed after 48 hours. After 4 hours of incubation with culture medium containing the CCK-8 reagent, the absorbance was read at 450 nm using a microplate enzyme-linked immunosorbent assay reader (Labsystems Dragon, Wellscan). Relatively inhibitory rate of cell growth was calculated according to the formula listed above. *R* = (*A*2 − *A*1)/*A*2 × 100% and *P* = *A*1/*A*2 × 100% in which *R* was relatively inhibitory rate and *P* was relatively proliferation ratio of cell growth; *A*1 was mean absorbance value of transfected cells and *A*2 was mean absorbance value of untransfected control cells without any drug treatment. All experiments were done with 5 wells per experiment and repeated at least three times.

### 2.6. Apoptosis Assay *In Vitro*

The cells were seeded in 6-well plates (4 × 10^5^/well). 12 h later, three dose concentrations of ZJW (obtained from the result of ZJW IC_10_ in CCK-8 assay) were added. Flow cytometry was used to detect apoptosis by determining the relative amount of AnnexinV-FITC-positive PI-negative cells, as previously described. Unstained cells, cells stained with Annexin V-FITC alone, and cells stained with propidium iodide alone were used as controls. Singly stained cells were used to adjust electronic compensation on FL1 and FL2 channels.

### 2.7. ICP-MS Analysis

The cells were harvested immediately after the end of drug exposure (L-OHP), washed twice with PBS, digestion with 0.25% trypsin, centrifuged at 12,000 ×g for 15 min at 4°C, and fragmentized for the sample processing. Ultrafiltrate samples (100 mL) were diluted using a Gilson ASPEC XLi programmed to deliver 1.8 mL of iridium internal standard (0.005 mg/mL, in 1% nitric acid). Samples were then mixed thoroughly prior to ICP-MS analysis. Intracellular L-OHP accumulation was determined by ultrasensitive multicollector inductively coupled plasma mass spectrometry (ICP-MS) as previously described.

### 2.8. Western Blot Analysis

Whole cell lysate for SDS-PAGE and Western blot analysis for P-gp, BCRP, MRP1/2, and LRP expression were prepared as previously reported [1]. The lysate was incubated on ice in immunoprecipitation assay buffer for 2 h before being homogenized using a mortar and pestle. The homogenized sample was centrifuged, and the supernatant was collected and stored at −80°C. Equal loading was confirmed with GAPDH (0.1 *μ*g/mL). Densitometric analysis was done using the Scion Imaging software (Scion Corporation), with GAPDH being a control for each sample.

### 2.9. MDR1 Promoter Activity by Vector Transient Transfection and Dual Luciferase Assay

The cells (2 × 10^4^ cells) were seeded in each well of 96-well culture plates in 100 *μ*L RPMI-1640 containing 10% fetal bovine serum and incubated at 37°C for 24 h in a 5% CO_2_-humidified atmosphere until the cells reached 90–95% confluence at the time of transfection. MDR1 promoter recombinant vector pGL3-Basic-MDR1 promoter (0.8 *μ*g/well) was mixed with a control vector (10 ng/well) pRL-SV40 in 25 *μ*L RPMI-1640 without fetal bovine serum and antibiotics. The solution was mixed with 0.5 *μ*L Lipofectamine 2000 reagent, diluted in 25 *μ*L RPMI-1640 without fetal bovine serum and antibiotics, and incubated at room temperature for 20 min; two vectors in 50 *μ*L solutions were cotransfected into each group cells after the cells were washed twice with RPMI-1640 without fetal bovine serum and antibiotics. The cells were incubated at 37°C for 12 h in a 5% CO_2_ humidified atmosphere. After transfection with plasmids, the medium was replaced with 100 *μ*L fresh RPMI-1640 without fetal bovine serum. 

After incubation overnight, the cells were washed with 100 *μ*L PBS and lysed by adding 20 *μ*L lysis buffer (Shanghai Lai'an Biotech. Co., Ltd., Shanghai, China). After incubation for 15 min at room temperature on a rocking bed (200 rpm), the lysate was centrifuged at 15,000 g for 5 min at 4°C and the supernatant was harvested and analyzed using a dual-luciferase assay kit (Shanghai Lai'an Biotech. Co., Ltd, Shanghai, China) as previously reported [1].

### 2.10. Statistical Analysis

All experimental data were expressed as mean ± standard of at least three independent experiments performed in duplicate. The statistical analysis was performed with the Student's *t*-test or covariance analysis for statistical significance. Statistical significance was set at a *P*-value of less than 0.05. All analyses were carried out using SPSS13.0.

## 3. Results

### 3.1. Quantitative Analysis of Various Active Compounds in the Extracts of ZJW

**Figure 1:**
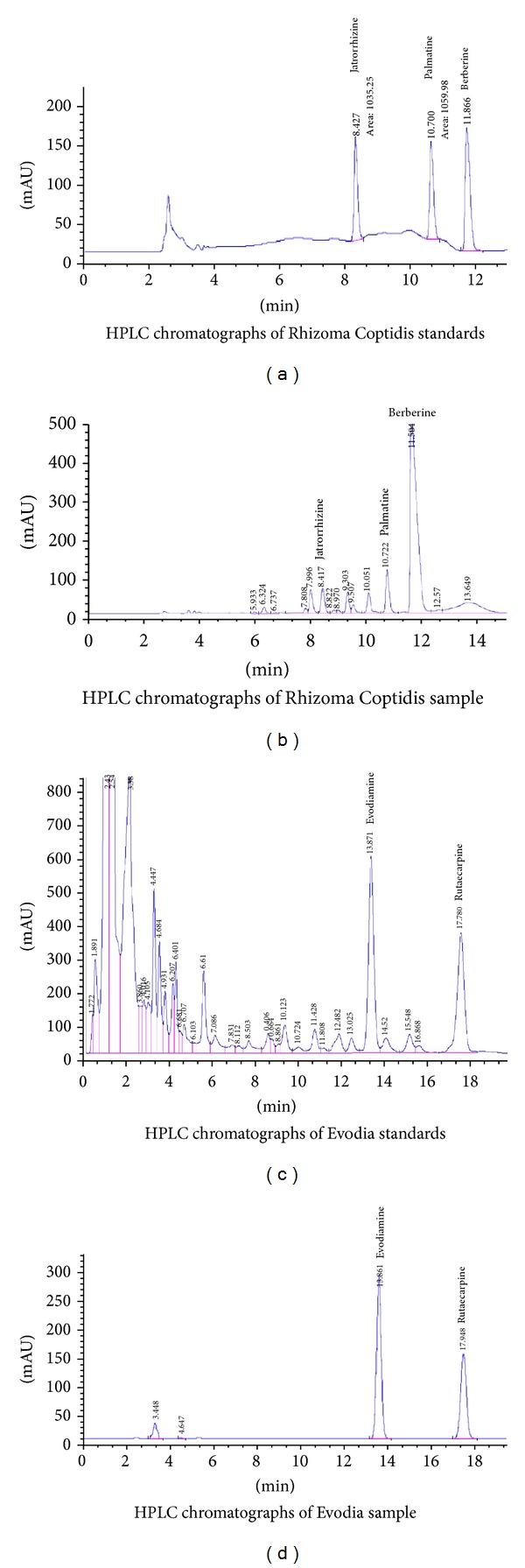
HPLC chromatographs of Rhizoma Coptidis and Evodia.

To ensure the quality and stability of ZJW solution, we used high-performance liquid chromatography (HPLC) to identify the components and confirm the final concentration of this solution. The resultant sample was analyzed on an Agilent 1100 HPLC system (Santa Clara, CA, USA) using a C-18 column (250 mm × 4.6 mm, 5 *μ*m). The mobile phase consisted of acetonitrile and water containing 0.05% formic acid. All analyses were performed at a column temperature of 30°C with mobile phase consisting of CH_3_CN : ddH_2_O (85 : 15, v/v) at a flow-rate of 1 mL/min with an injection volume of 10 *μ*L. The detection wave length was set to monitor at 225 nm. As shown in Figure 1(a), quantitative results of jatrorrhizine, palmatine, and berberine in Rhizoma Coptidis were 3.8%, 6.4%, and 57.1%, respectively. As shown in Figure 1(c), quantitative results of evodiamine and rutaecarpine in Evodia were 2.29% and 3.18%, respectively. Various components in ZJW extracts were determined by referring to the calibration curve established by the running standard at varying concentrations under the same conditions (Figures 1(b) and 1(d)).

### 3.2. Noncytotoxic Dose of ZJW

**Figure 2:**
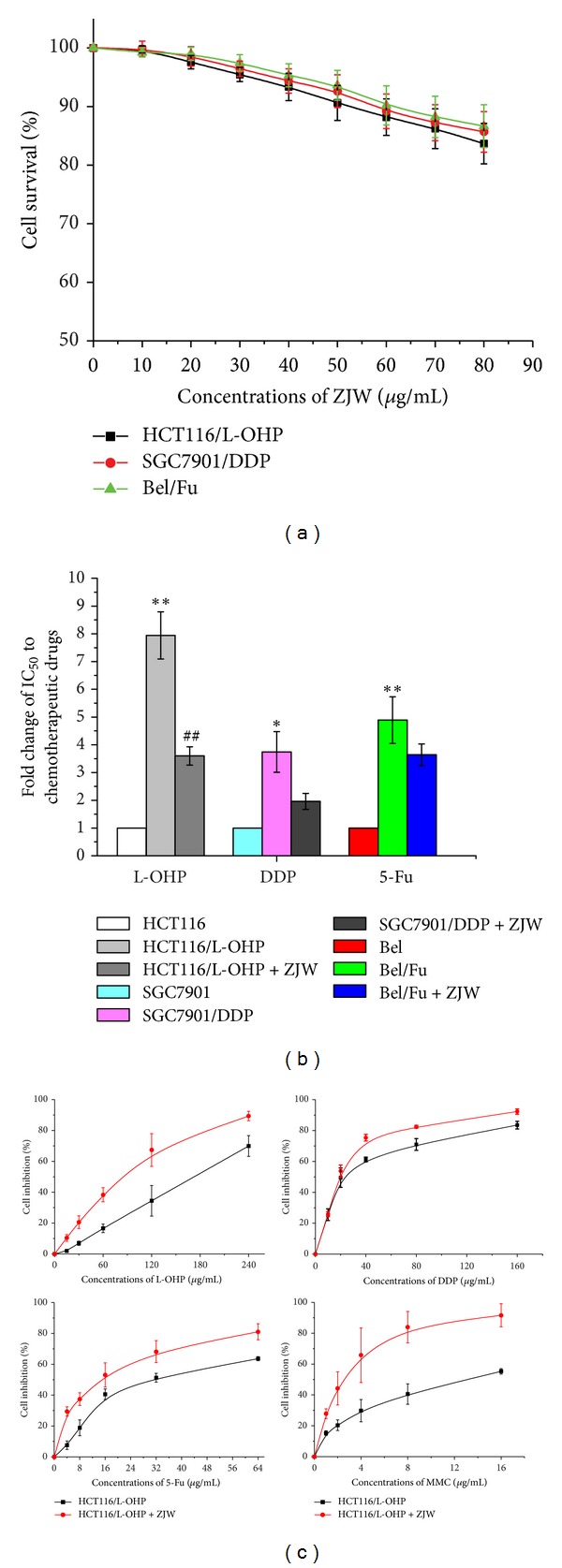
Effect of ZJW on the sensitivity to chemotherapeutic drug in human cancer cells. (a) The cells were treated with various concentrations of ZJW and analysis by CCK-8 analyses. (b) Antiproliferative IC_50_ values of L-OHP, DDP, and 5-Fu on HCT116/L-OHP, SGC7901/DDP, and Bel/Fu cells were analyzed by CCK-8 analyses. Data are presented as mean ± SD of triplicate experiments. *P < 0.05,  **P < 0.01,  ^#^ *P* < 0.05, and ^##^ *P* < 0.01 versus control. (c) CCK-8 assay was used to detect cell inhibition of L-OHP, DDP, 5-Fu, and MMC in HCT116/L-OHP cells treated with ZJW at 50 *μ*g/mL for 48 h.

Previous work from this lab has shown that ZJW is a potent drug with the effect of anti-cancer in solid tumor cells, such as human colon carcinoma and pancreatic cancer cells [22]. In order to avoid the possibility that inhibition of cell proliferation is due to cytotoxicity, we first examined the cytotoxic effect of ZJW on different cancer cell lines, including colorectal cancer cell (HCT116/L-OHP), gastric cancer cell carcinoma (SGC7901/DDP), and hepatic carcinoma cell (Bel/Fu). According to the permitted standardization of the noncytotoxic dose, in our study, data showed that the dosage of IC_10_ in HCT116/L-OHP cell was 50 *μ*g/mL, 54 *μ*g/mL in SGC7901/DDP cells and 60 *μ*g/mL in Bel/Fu cells (Figure 2(a)). Less than these dosages, cell survival was not found to be significantly different from untreated cells (100% survival). Therefore, for all cell proliferation experiments, the cells were treated with ZJW in the concentration range of 50 *μ*g/mL, which is the lowest dosage of the IC_10_ in the three cell lines.

### 3.3. Effect of ZJW on the Sensitivity to Chemotherapeutic Drug in Human Cancer Cell

To determine the best effect of ZJW on the sensitivity to chemotherapeutic drug in human cancer cell lines, all cell lines were treated for ZJW with their own induced chemotherapeutic drug and then analyzed by CCK-8 assay with IC_50_ of L-OHP, DDP, and 5-Fu. As shown in Figure 2(b), ZJW displayed significant cytotoxicity in HCT116/L-OHP cell, with an IC_50_ value from 157.48 ± 16.73 *μ*g/mL to 71.40 ± 6.48 *μ*g/mL, while the IC_50_ against the parental cells was 19.84 ± 1.42 *μ*g/mL. Notably, the resistance effect of ZJW in SGC7901/DDP cells and Bel/Fu cells was less than that of HCT116/L-OHP cell. Therefore, our results suggested that ZJW owned the best effect on synergistic therapy in human colorectal cancer.

### 3.4. Increasing Effect of ZJW on the Sensitivity to Chemotherapeutic Drug in HCT116/L-OHP Cell

In order to confirm the effects of ZJW on proliferation in HCT-116/L-OHP cells, we tested the inhibition in MDR cells after cells treatment with 3 different kinds of chemotherapeutic drugs. As the result suggested, HCT-116/L-OHP cells were more resistant to L-OHP, DDP, 5-Fu, and MMC respectively in a dose dependent manner (Figure 2(c)). Therefore, these data further suggest that ZJW was responsible for increasing the sensitivity to chemotherapy to reverse MDR in HCT-116/L-OHP cells.

### 3.5. ZJW-Induced Apoptosis without Affecting Cell Cycle Distribution of HCT-116/L-OHP Cells

**Figure 3:**
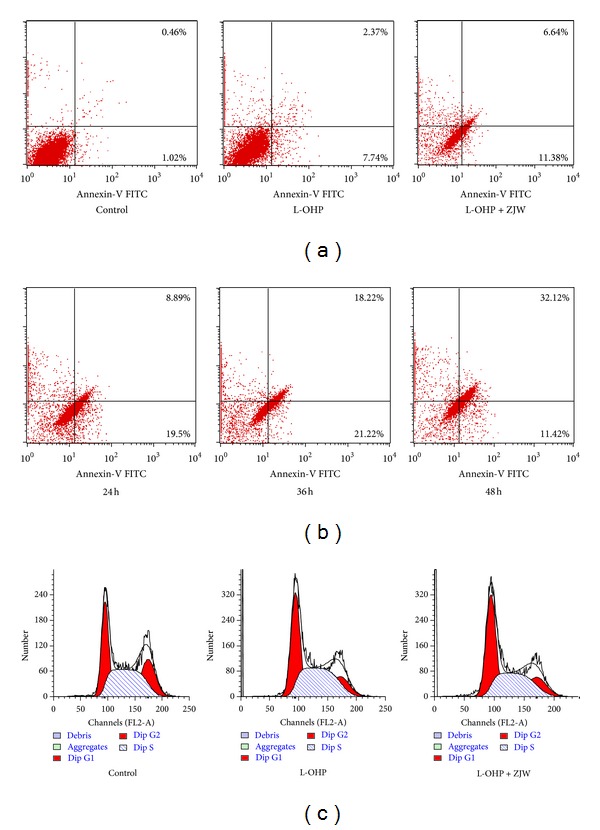
Effect of ZJW on apoptosis and cell cycle in HCT116/L-OHP cells. (a) Flow cytometry analysis of apoptosis with Annexin V-FITC/PI binding to HCT116/L-OHP cells. Viable cells (Annexin V^−^/PI^−^) are located in the lower left, apoptotic cells (Annexin V^+^/PI^−^) in the lower right, postapoptotic secondary necrotic cells (Annexin V^+^/PI^+^) in the upper right, and primary necrotic cells (Annexin V^−^/PI^+^) in the upper left quadrants, respectively. Numbers in each quadrant are percentages of cells. L-OHP group means HCT116/L-OHP cells exposure of L-OHP for 24 h; L-OHP + ZJW group means HCT116/L-OHP cells exposure of L-OHP and ZJW (50 *μ*g/mL) for 12 h and HCT116/L-OHP cells without any treatment as the control. (b) Flow cytometry analysis of apoptosis with Annexin V-FITC/PI binding to HCT116/L-OHP cells after treatment with ZJW at 24 h, 36 h, and 48 h. (c) Flow cytometry analysis of cell cycle distribution in three groups which described as (a).

To further investigate the mechanisms underlying the inhibiting proliferation effect of ZJW, Annexin V and propidium iodide (PI) double staining was performed to see the change of cell apoptotic induced by L-OHP after treatment with ZJW. The results showed that ZJW did not influence the HCT-116/L-OHP cell distribution in normal (both annexin V and PI negative) and early apoptosis (annexin V positive and PI negative), but significantly increase the late apoptotic or necrotic populations (both annexin V and PI positive) (Figure 3(a)). To further examine whether the effect of ZJW on cell apoptosis occurred in different time, cell apoptosis induced by L-OHP was determined at ZJW treatment for 24 h, 48 h, and 72 h. As shown in Figure 3(b), exposure to ZJW also increased L-OHP-induced cell apoptosis in a time-dependent manner. However, the cell cycle analyses obtained from the Flow cytometric analysis showed that there was no change in any phase arrest in response to treatment with ZJW compared with control group (Figure 3(c)). All of these observations suggest that ZJW did not alter cell cycle in HCT-116/L-OHP cells. These data suggest that the reversal effect of ZJW was most likely obtained by direct induced apoptosis as opposed to later the cell cycle.

### 3.6. Effect of ZJW on Intracellular L-OHP Accumulation

**Figure 4:**
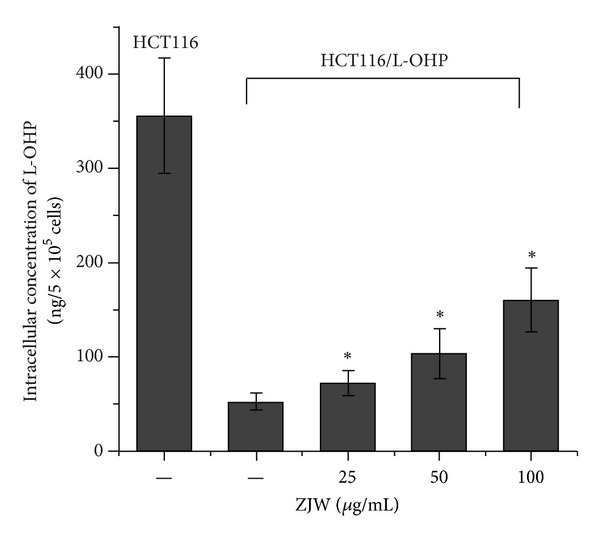
Effect of ZJW on intracellular L-OHP accumulation. A validated ICP-MS method was used to detect intracellular L-OHP accumulation in HCT116 and MDR HCT116/L-OHP cells which were treated with ZJW at 25 *μ*g/mL, 50 *μ*g/mL, and 100 *μ*g/mL for 48 h, meanwhile HCT116 cells were taken as control group. The data are representative of at least three experiments, which are presented as the mean ± SD. *P < 0.05 ZJW (25 *μ*g/mL, 50 *μ*g/mL and 100 *μ*g/mL) versus ZJW (0 *μ*g/mL).

To determine whether ZJW causes an increase on the intracellular chemotherapeutic drug accumulation in colorectal cancer cell, we evaluated the effect of ZJW on concentration of L-OHP in HCT-116/L-OHP by ICP-MS assay. As shown in Figure 4, the intracellular L-OHP accumulation in HCT-116/L-OHP cells was significantly lower than it was in HCT-116 cells. After 48 h treatment of ZJW with different dosage, intracellular L-OHP accumulation in HCT-116/L-OHP cells was increased in a concentration-dependent manner. As the data suggested, ZJW at a concentration of 25 *μ*g/mL had a little effect of increasing chemotherapeutic drug accumulation, but significantly increased intracellular L-OHP accumulation at the concentration of 100 *μ*g/mL. These results showed that ZJW significantly increased chemotherapeutic drug accumulation in a dose-dependent manner in HCT-116/L-OHP cells.

### 3.7. Effect of ZJW on the Level of P-gp, BCRP, MRP1/2, and LRP *In Vitro*

**Figure 5:**
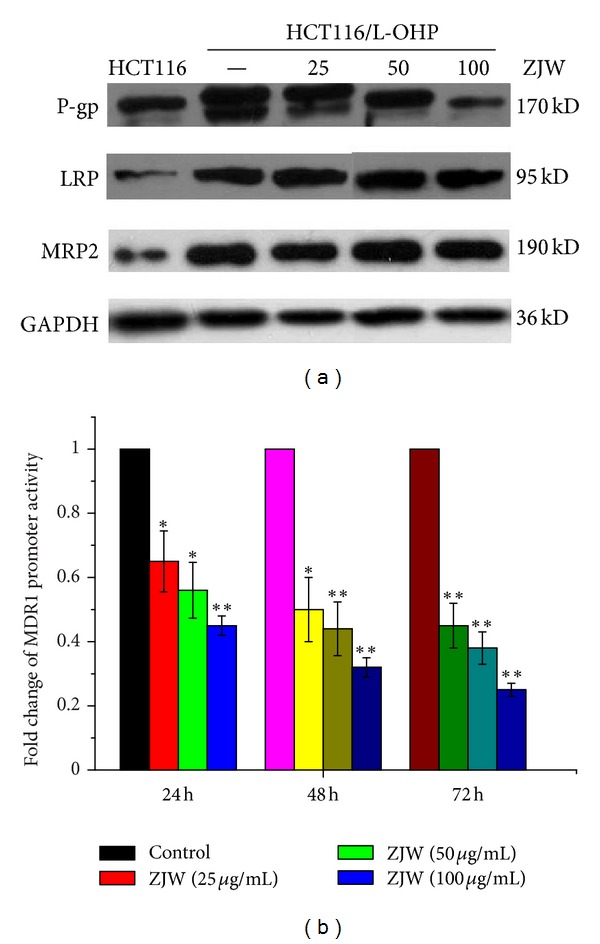
Inhibition effect of ZJW on the ABCB1 gene and its encoded P-gp *in vitro*. (a) Western blotting assay was carried out to detect the level of P-gp, LRP, and MRP2 in HCT116 cells, HCT116/L-OHP cells, and HCT116/L-OHP cells treated with ZJW for at 25 *μ*g/mL, 50 *μ*g/mL, and 100 *μ*g/mL for 48 h, respectively. Western blotting with an antibody to GAPDH was used to ensure equal loading of proteins in each lane. The bolts were photographed and quantitated for each sample; the data are from three independent experiments. (b) The activity of MDR1 promoter in HCT116/L-OHP cells treatment with ZJW at 24 h, 48 h, and 72 h was analyzed by dual-luciferase assay kit. The resulted data is firefly luciferase/renilla luciferase from different groups. Data are means ± standard deviation of values from triplicate experiments. *P < 0.05, **P < 0.01 represents that ZJW treatment group is significantly different from control group at 24 h, 48 h, and 72 h.

To further investigate the mechanisms underlying the resistant effect of ZJW on colorectal cancer MDR cells, we determined the effect of ZJW on the levels of the four cell membrane-bound ATP binding cassette (ABC) transporters in HCT-116/L-OHP cells, which is P-gp, BCRP, MRP2, and LRP. As shown in Figure 5(a), the incubation of cell with ZJW (25, 50, and 100 *μ*g/mL) for 48 h did not significantly alter the level of MRP2 and LRP; however, the level of P-gp was significantly decreased at a concentration-dependent manner in HCT116/L-OHP cell. In addition, Western blot analyses results also indicated that there was no expression of MRP1 and BCRP in the MDR cells (data not shown). This suggests that the MDR reversal effect of ZJW in HCT116/L-OHP cells may be dependent of the inhibition on the level of P-gp.

### 3.8. Effect of ZJW on the Activity of MDR1 Promoter *In Vitro*

To further ascertain whether the effect of ZJW on MDR1/P-gp expression occurred at the level of transcription, the activity of MDR1 mRNA was determined by the recombinant vector pGL3-MDR1-promoter which has been transfected into HCT116/L-OHP cells. We observed a downregulation in the expression of MDR1 promoter in HCT116/L-OHP cells treatmed with ZJW at 24 h, 48 h, and 72 h. In addition, our data clearly show that ZJW could inhibit the activity of MDR1 promoter in a dose-dependent manner (Figure 5(b)). Considered together, our findings imply that the reversal effect of ZJW on MDR1/P-gp mediated MDR in HCT116/L-OHP cells is by influencing the transcription and translation from MDR1gene to P-gp expression.

### 3.9. ZJW Reverses MDR in Nude Mice Xenograft Model

**Figure 6:**
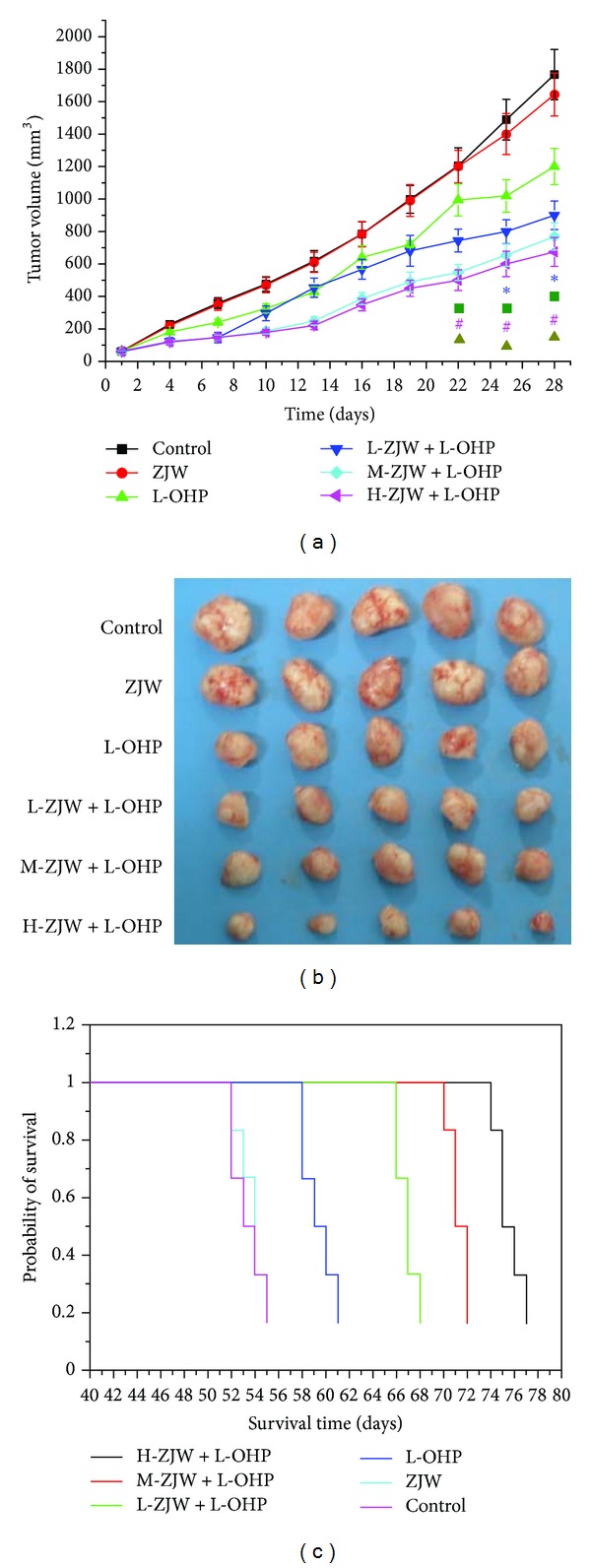
Inhibition effect of ZJW *in vivo*. (a) Tumor volume change was determined every two days during delivery period. Values are mean weight of nude mice ± SD. Statistical difference was analyzed by Student's *t*-test. *P < 0.05 represents that L-OHP (5 mg/kg) treatment group is significantly different from control group; ^■^ *P* < 0.05 represents that L-ZJW (1027.5 mg/kg) + L-OHP (5 mg/kg) treatment group is significantly different from control group; ^#^ *P* < 0.05 represents that M-ZJW(2055 mg/kg) + L-OHP (5 mg/kg) treatment group is significantly different from control group; ^▲^ *P* < 0.05 represents that H-ZJW(4110 mg/kg) + L-OHP (5 mg/kg) treatment group is significantly different from control group. (b) Tumors removed from nude mice and photographed on the 28th day after administration. (c) Overall survival of xenograft model after injection with HCT116/L-OHP cells.

To explore whether ZJW reverses cancer MDR *in vivo*, we employed a colorectal MDR cancer xenograft model. The tumor volume and body weight were observed in the six groups, respectively. There was no significant difference in tumor size between animals treated with saline, or ZJW, indicating the anti-cancer effect of ZJW is not obvious *in vivo*. However, the combination of L-OHP and ZJW produced a significant inhibition of tumor growth compared with animals treated with saline or L-OHP alone (*P* < 0.05). As shown in Figure 6(a), ZJW helped L-OHP inhibit the mice tumor volume at a concentration-dependent manner. Furthermore, we also measured the difference in survival times among these groups. The control group began to die at 52 days and all succumbed to disease by 55 days (Figure 6(b)). In comparison, the first mouse from the H-ZJW + L-OHP group died in 74 days, and the last two in the group died after 77 days. The results suggest a significant increase in the survival time (*P* < 0.01) with synergy effect of ZJW, although there were no animals that ultimately survived the disease.

### 3.10. ZJW Reverses P-gp-Mediated MDR *In Vivo*

**Figure 7:**
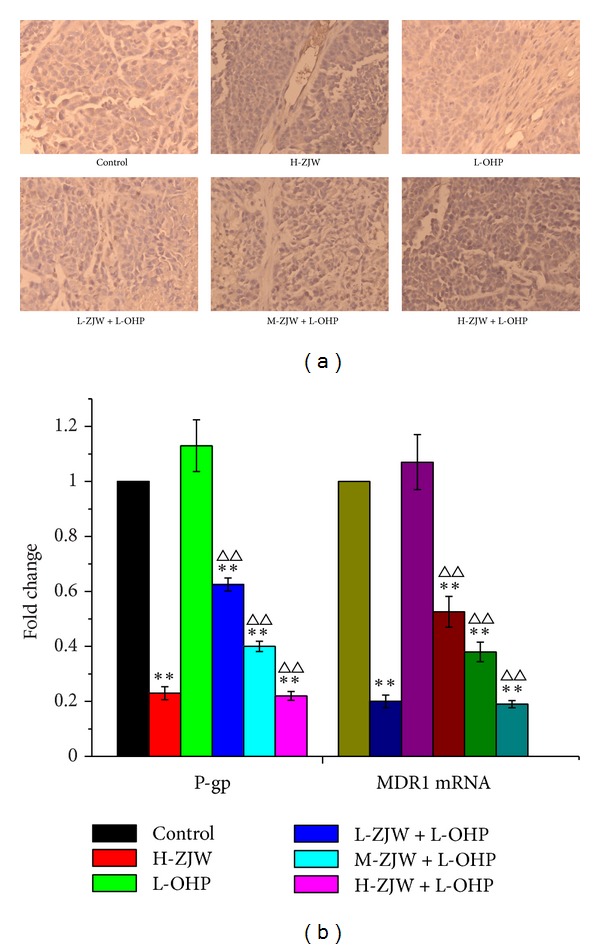
Inhibition effect of ZJW on the level of P-gp and the expression of MDR1 mRNA *in vivo*. (a) The xenograft tumor tissue in mice of all groups was subjected to immunohistology analysis using P-gp antibody; the results were magnified 200 in microscope; positive cells were brown staining; negative cells were blue staining. (b) The xenograft tumor tissues in mice of all groups were subjected to real-time qPCR to determine the mRNA expression of MDR1. Quantification of P-gp and MDR1 mRNA was performed by assigning a value of 1 to the group treated with H-ZJW (4110 mg/kg) + L-OHP (5 mg/kg). Statistical difference was analyzed by Student's *t*-test, **P < 0.01 versus control group; **P < 0.01 versus L-OHP alone group. This is a representative result of three repetitive experiments with similar results.

To confirm whether the synergy effect of ZJW on xenograft tumor growth was mediated by decreasing the level of P-gp, we investigated the expression level of P-gp in xenograft tumor of mice treated with combination of L-OHP and ZJW by immunohistology analysis. As shown in Figure 7(a), the level of P-gp is decreased in xenograft tumor of mice treated with ZJW and L-OHP compared with that in control group. 

Real-time qPCR was also used to investigate the mRNA expression of MDR1 which is P-gp target gene in xenograft tumor treated with ZJW and L-OHP. Following the treatment of ZJW, there was a significant decline of the expression of MDR1 mRNA in ZJW group compared with that in control group. Moreover, the significant decline of MDR1 mRNA was observed in the combination of L-OHP and ZJW than that in L-OHP alone (Figure 7(b)). Therefore, the results *in vivo* further confirmed that the anti-MDR effect of ZJW was partly mediated by decreasing the level of MDR1/P-gp.

## 4. Discussion

Over a period of time, MDR is a critical problem that continues to hamper the success of modern chemotherapy against cancer [23]. It is a complicated multifaceted result, and it is mediated by a series of integral membrane proteins, including ABCB1/P-gp, ABCC-1/2, ABCG2/BCRP, and LRP. To reverse chemotherapeutic drugs-mediated MDR, numerous studies have attempted to develop some more effective chemotherapeutic drugs [24–26]. However, the tolerance of chemotherapeutic drugs still exists; even modern medicine continues to develop and create in some studies. Moreover, although many clinical trials have been conducted for some specific targets, most results have been disappointing, and the toxicity of these modern medicine themselves is one important factor that led to the failure of these studies [27].

Since it has been a critical problem of chemotherapy with poor effect in the treatment of cancer, the development of anti-MDR agents has become a major focus on overcoming cancer drug resistance. Traditional Chinese prescriptions and formulae, as a major constituent of several herbal products, represent an ideal compound for reversing MDR due to its low toxicity. Previous studies have confirmed that ZJW has potent anti-cancer and synergistic effects by inhibiting the growth of S180 tumor *in vivo* [28]. Recent findings have found that berberine and coptisine, which are the major active constituents of Coptis, were found to reverse ABCB1-mediated MDR in human MDR cancer cells [14, 15].

In order to elucidate its anti-cancer molecular mechanisms, the present research focused on the effects of ZJW ethanol extracts in reversing MDR. In our present study, the indicator components of ZJW extract including Rhizoma Coptidis and Fructus Evodiae have been detected by HPLC/ESI-MS analysis. To investigate the anti-MDR effects of ZJW on human cancer, HCT116/L-OHP, SGC7901/DDP, and Bel/Fu cells growing exponentially were treated with ZJW (0–600 *μ*g/mL), and the IC_10_ of ZJW was measured by CCK-8. In the three MDR cell lines, the survival at the IC_10_ was not found to be significantly different from untreated cells, and it permitted standardization of the noncytotoxic dose. These results suggest that concentrations of ZJW below 50 *μ*g/mL are not toxic to the three MDR cells. Therefore, for all cell proliferation experiments, the cells were traded by ZJW in the concentration range of 0–50 *μ*g/mL. The MDR cells used in cell proliferation experiments at a noncytotoxic dose are similar to those reported previously for U251 cancer cells, which were treatmented with a noncytotoxic dose [29]. Furthermore, CCK-8 analyses were performed in HCT116/L-OHP, SGC7901/DDP, and Bel/Fu cells to determine their relative ability in increasing, concentrations of the chemotherapy drugs, L-OHP, DDP, and 5-Fu. Data showed that HCT-116/L-OHP cells are much more resistant to death induced by L-OHP after ZJW treatment when compared with the other cells. Because colorectal cancer cell displayed the maximum degree of downregulation in IC_50_ of chemotherapeutic drugs, we used colorectal cancer for the next experiment assays. In addition, ZJW significantly increased the cellular toxicity of L-OHP, DDP, 5-Fu, and MMC in HCT-116/L-OHP cells and greatly enhanced the inhibition rate of chemotherapeutic agents in a dose-dependent manner.

In our study, we found ZJW had the synergistic effect in chemotherapeutic drugs induced-cell apoptosis in a time-dependent manner, as several previous reports also have shown the synergy effect of traditional Chinese prescriptions and formulae [30, 31]. However, we did not observe obvious alterations in any cell cycle arrest of HCT-116/L-OHP cells after treatment with the formula. One possible explanation is that there might be interaction between different herb constituents of this formula which could lead to complicated effects. Another factor is that ZJW could not alter L-OHP-induced cell cycle arrest since it is considered L-OHP as a noncycle specific antineoplastic agents. These findings are in accordance with some reports [32, 33], which show opposite roles for some drugs in tumor development and cause apoptosis but not cell cycle alterations.

To elaborate the reversal ability of ZJW, we investigated the effect of ZJW on the accumulation of L-OHP in HCT116/L-OHP cells. Based on these data by ICP-MS analysis, we have shown that ZJW remarkably enhanced the intracellular accumulation of L-OHP in a dose-dependent manner. These results were in accordance with those of the CCK-8 assay, which altogether proved that ZJW was able to increase the sensitivity of MDR cells to chemotherapeutic agents.

Other studies showed that targeted agents or inhibitors/modulators of efflux transporters could inhibit energy-dependent efflux of protein on the cell membrane and cause transport mechanism of membrane protein in human MDR cancer cells [34]. Earlier work from this laboratory demonstrated a correlative relationship between signal transduction pathway and P-gp in the colorectal MDR cell [1]. Here we have extended these studies using three human MDR cell lines and detected the effect of ZJW on the expression of ABCB1/P-gp, ABCC-1/2, ABCG2/BCRP, and LRP. We found that ABCB1/P-gp, ABCC-2, and LRP were more upregulated in HCT116/L-OHP than in HCT116 cells. Further analysis revealed that ZJW-reverse MDR was accompanied by the downregulation of P-gp, but not through ABCC-2, and LRP-mediated MDR pathway. It suggests that ZJW-reversed MDR may be dependent on the inhibition of ABCB1/P-gp. Our data support the suggestion by Chao et al. about the link between ZJW and TPA-induced AP-1 activities by altering anchorage-independent cellular growth in a concentration-dependent manner [35]. Because it is known now that there are AP-1 binding sites on the promoters of ABCB1 blockage of tumor promoter-induced AP-1 activation inhibits neoplastic transformation. Therefore, the reversal effect of ZJW may be regulated through the inhibition of AP-1 mediated P-gp.

In light of our *in vitro* data on the effect of ZJW in human colorectal MDR cancer cells to chemotherapeutic drugs, we examined the *in vivo* therapeutic potential of ZJW. Indeed, animal experiments results showed that the anticancer effect of ZJW on resistant cancer cells xenograft is better than that of L-OHP control group. In this study, we provided evidence that combination of chemotherapy with herbal medicine formula ZJW prolonged the overall survival time of xenograft model. And these results demonstrated that ZJW exhibited a good downregulation on the expression of ABCB1/P-gp both *in vitro *and *in vivo*.

In conclusion, our data strongly imply that ZJW inhibited P-gp-mediated MDR both *in vitro* and *in vivo*. First, ZJW reversed MDR via increasing the sensitivity of MDR cells to chemotherapeutic agents. Second, ZJW reversed MDR through down-regulation of P-gp *in vitro* and *in vivo*. And third, combination of chemotherapy with ZJW prolonged the overall survival time of xenograft model and reduced the tumor volume. Thus, this study has provided a natural potent inhibitor of human MDR. Compared with modern medicine, combination of therapeutic principles represented by multiple herbs may yield better results in cancer treatment. ZJW, a traditional Chinese herbal medicine, has been demonstrated to have implications for the rational development of novel regimens in human MDR.
